# Different impact of factor VIII products on inhibitor development?

**DOI:** 10.1186/s12959-016-0102-4

**Published:** 2016-10-04

**Authors:** H. Marijke van den Berg

**Affiliations:** Julius Center for Health Sciences and Primary Care, University Medical Center, Utrecht, The Netherlands

**Keywords:** FVIII clotting products, Inhibitor development, Severe hemophilia A PUPs

## Abstract

Inhibitors are the most serious side effect of haemophilia treatment; they occur in 25–30 % of all patients with severe haemophilia A. Over the last 2 decades, conflicting data on the impact of clotting products have been published.

Due to small studies of selected cases, appreciation of the impact of any particular product has been difficult. Moreover, the emphasis on inhibitor testing has led to increased detection of low-titre inhibitors (to >10 %), while the percentage of high-titre inhibitors is still around 20 %.

Other non-genetic risk factors, such as dosing and intensive treatment, are able to increase individual inhibitor risk. Early prophylaxis might reduce inhibitor risk. Well-defined large PUP studies including products should be considered. This can only be achieved in collaboration with all stakeholders.

In conclusion, while the impact of FVIII products on inhibitor development is large, presently the actual impact of any specific product is unclear.

## Background

The development of inhibitory antibodies in severe haemophilia A is the major side effect of treatment. In patients with severe haemophilia A, 25–30 % will develop antibodies [1, 2]. From the start, the direct correlation between the infusion of factor VIII and the development of these allo-antibodies has clearly linked factor VIII products to inhibitors. The risk to develop inhibitors is directly correlated to the number of exposure days; they develop mainly in the first 50 exposure days (EDs). Especially during the first 20 EDs there is a steep rise, with 50 % of the inhibitors having developed at 14–15 EDs [1, 2].

The availability of clotting products has an important impact on treatment strategies and the age prophylaxis is started. Plasma products were scarce in the seventies and eighties and prophylaxis was only introduced in countries such as Sweden and The Netherlands [3, 4]. Over the years these countries recognised that an early start was crucial and they started prophylaxis at 1–2 years. In countries still using episodic treatment, inhibitors were often diagnosed in adults after many years of treatment. In the eighties with the fear of HIV, inhibitors did not gather much attention, as physicians and patients were very careful in their dosing and in many countries prophylactic treatment was still not introduced [5]. After the large problems with viral pathogens transmitted through plasma products, the introduction of recombinant products in the early nineties made it possible to treat a new generation of children with haemophilia with recombinant products without the fear of transmitting pathogens. In the early nineties, an outbreak of inhibitors occurred in adult patients who were treated for more than 150 EDs [6, 7]. This unusual event urged the regulators to ask for stringent studies in previously untreated children (PUPs). In the past no formal PUP studies with locally produced plasma products were performed. In clinical trials frequent testing for inhibitors was mandatory. Single positive laboratory tests in a patient were reported as inhibitors [8]. The high inhibitor results in the first PUP studies with recombinant products led to the discussion whether a higher risk for inhibitors existed with recombinant products as compared to plasma products. In the next paragraph the most important factors influencing the comparison of different studies are discussed.

## Review

### Risk factors for inhibitor development

In the last decade many new risk factors for inhibitor development have been recognised. It is now commonly accepted that inhibitor development is influenced by multiple risk factors [9]. In general, these factors can be divided in genetic and non-genetic risk factors. After genetic analysis of the *F8* gene became available, a clear correlation was demonstrated between high-risk *F8* gene mutations and inhibitor development [10, 11]. The importance of the causative mutation is further established in a recent meta-analysis in severe haemophilia A by Gouw et al. [12]. This confirmed that the inhibitor risks in patients with large deletions were five times higher than for nonsense mutations. However patients with intron 22 inversions had an average risk.

Another study that shed new light on the impact of the FVIII gene was the Insight study [13]. In this large international study including patients with mild haemophilia, the overall risk for inhibitors clearly increased in patients with more EDs. The inhibitor risk doubled between 50 and 100 EDs to > 13 %, which is much higher than previously reported [13].

Afro-American ethnicity was correlated with up to 50 % risk; but this needs to be confirmed in larger studies [11].

Studies on polymorphisms in immune regulatory genes explored a more mechanistic approach between genetic and non-genetic risk factors [9]. Especially T-cell responses are important, but their exact role has been difficult to establish due the small number of inhibitors in studies.

### Products

The best studied non-genetic risk factor is the product used for treatment during the first 50EDs. Wight and Paisley concluded in their 2003 review that the risk for plasma products was lower [14]. From that time onwards, many meta-analyses were performed on newly published studies [15–17]. Overall, a lower risk for class plasma products was concluded, although the risk difference decreased over the years [17]. Several academic groups recognised the shortcomings in the different study designs and the importance to collect large amounts of data from many different products [18, 19]. Large observational cohort studies using the same definition for inhibitors did not confirm a difference in inhibitor risk between class plasma and class recombinant products [2, 19].

A very important non-genetic risk factor is the effect of high dosing and intensive treatment [20–24]. Studies have demonstrated that inhibitor risk can increase trice during periods of peak treatment. In the meantime, the increased availability of products and the small vial sizes of recombinant products made it much easier to increase dosing in small children.

The potential role of von Willebrand factor (VWF) as an immune protective chaperone for FVIII is not clear, but it may act through antigenic competition and/or by reducing endocytosis of the FVIII molecule in a dose-dependent manner, thereby preventing activation of immune effectors [24].

An unexpected observation was a higher inhibitor risk for a specific second generation recombinant product [19]. This observation was confirmed in two other independent studies [25, 26]. The strengths of these studies are that they included all eligible patients and followed them until at least 50EDs and that they all used the same definition for clinically relevant inhibitors. This gives a more appropriate overall risk and enables comparison between products. A weakness is that not all inhibitor samples are confirmed in a central laboratory, which could lead to misdiagnosis in low-titre inhibitors.

To give a definitive answer about whether there is a risk difference between class plasma and class recombinant products, the SIPPET study was performed a randomised controlled study between class plasma and class recombinant products in circa 250 PUPs with severe haemophilia A [27]. It was concluded that plasma products had a significantly lower risk than recombinant products [27]. Although the RCT design is the most preferable one for comparison of 2 products or 2 strategies, it is debatable what the outcome of this study means for the current choice of products.

The first non-genetic risk factor that was determined was the age at first exposure to FVIII [28]. Start of treatment before 6 months of age significantly increased the risk. This association could be largely explained by early, intensive treatment for major bleeds and surgery [2, 29]. Another very interesting observation was the lower inhibitor risk in patients who received prophylaxis from an early age onwards [2, 29]. During prophylactic treatment, patients are exposed to factor VIII in the absence of intensive treatment and tissue damage. While several studies have confirmed the effect, the difference in study design and the short period until inhibitor development presently hinders a definitive conclusion regarding the preventive effect of prophylaxis [30].

### Impact of inhibitor testing

The definition of inhibitors was historically based on the lack of response to treatment. Bleedings were unresponsive and the clinical symptoms pointing at an inhibitor were confirmed by a positive Bethesda assay. After the introduction of recombinant products and the results of the clinical PUP studies that reported inhibitors in 25–35 % of PUPs, regular testing to detect inhibitors became the norm. Nowadays it is recommended to test at least every 3 months for inhibitors during the first 50 EDs [31]. In a large study, the PedNet group divided all PUPs according to 5-year birth cohorts [32]. Over a 20-year period, inhibitor titres over a 20-year period where investigated in 926 PUPs with severe haemophilia. The strength of the study is that the same definition of inhibitors is used and that all patients were followed until 50 EDs. The total inhibitor incidence increased from 19.5 % in 1990–1994 to 30.9 % in 2000–2004 (p 0.011). This was solely due to the detection of more low-titre inhibitors, increasing from 3.1 % to 10.5 % (*P* = 0.009) [32].

## Discussion

Inhibitor development is currently the most serious side effect of the treatment with clotting factor products. It is caused by the interplay of many risk factors [9]. Since inhibitors only develop after treatment has been started, a direct correlation with the type of clotting product was expected. In the early 1990s the awareness for inhibitors has increased after the outbreak of inhibitors on certain plasma products [6, 7]. At the same time recombinant products were registered according to stringent procedures, and authorities demanded frequent testing for inhibitors [8]. The coincidence of these occurrences has probably led to the assumption of a higher risk for recombinant products.

To rule out any confounding factors, the SIPPET study was performed [27]. This study found a higher risk for recombinant products. Since the study was performed mostly in other geographical areas and was prematurely stopped, the impact on future treatment choice is unclear. EMA and FDA have taken the results seriously and requested to review all data (http://www.ema.europa.eu/docs/en_GB/document_library/Referrals_document/Factor_VIII_31/Procedure_started/WC500209984.pdf). From a pharmacological point of view it is doubtful if a comparison between class products will lead to clinically meaningful results. Products are all different and the effects of any product should be investigated separately.

Factors that may influence the results of studies are numerous. The most important factors are the small numbers of subject in many studies. It is clear that at the time of marketing authorisation, the safely profile of clotting products is not completely clear. Especially for immunogenicity, large data in PUPS needs to be collected. The World Federation of Hemophilia has acknowledged this and introduced templates for data collection to be used by haemophilia centres [33]. It is especially advisable that centres collect data on all PUPs with severe and moderate haemophilia and share the results. There is even a larger need with all the new products that will be marketed. Independent comparison of products is only possible when the data collected is similar and includes all confounding factors.

The importance of central testing is clear; it can largely reduce laboratory variations. It has to be weighed against missing samples in small children, which will affect the outcome of studies as well. The emphasis has been too much on the single laboratory tests. Other factors should be included as well, such as reduced recovery and clinical signs of bleeding. The definition of a clinical important inhibitor, excluding single positive laboratory tests, has been proven to be of great importance. Overall more low-titre inhibitors have been detected [8, 32]. Interestingly, many of the on-going large prospective observational studies, despite not having performed central testing, still have found very similar results for high-titre inhibitor incidences [2, 18, 19].

## Conclusion

In conclusion, inhibitor development is a major side effect of treatment. All current products have an unacceptable high risk for inhibitors and large international collaborations are needed to Improve the mechanism of inhibitor formation.

### Key points

- High titre inhibitors should be the primary outcome
- Currently all factor VIII products have a large risk of inhibitor development
- Further studies are needed to confirm a lower risk for any particular FVIII product
- Large international collaborations between all stakeholders should address the inhibitor problem
